# Effects of rope skipping exercise on working memory and cardiorespiratory fitness in children with attention deficit hyperactivity disorder

**DOI:** 10.3389/fpsyt.2024.1381403

**Published:** 2024-05-23

**Authors:** Ziyun Huang, Liang Li, Yijuan Lu, Jie Meng, Xueping Wu

**Affiliations:** ^1^ School of Athletic Performance, Shanghai University of Sport, Shanghai, China; ^2^ School of Physical Education, Shanghai University of Sport, Shanghai, China; ^3^ Shanghai Shiguang School, Shanghai, China; ^4^ School of Sports and Health, Linyi University, Linyi, China

**Keywords:** attention deficit hyperactivity disorder, working memory, cardiorespiratory fitness, exercise, children

## Abstract

**Introduction:**

Children with Attention Deficit Hyperactivity Disorder (ADHD) exhibit deficits in working memory (WM) and cardiorespiratory fitness (CRF), both of which are closely associated with the core symptoms of ADHD. This study aimed to investigate the effects of rope skipping exercise (RSE) on the WM and CRF of children with ADHD, to provide a theoretical foundation for the optimization of exercise intervention programs tailored to children with ADHD.

**Methods:**

This study recruited 55 children (age range 6–12 years) and randomly assigned them into three groups: the ADHD with RSE (AWRSE, n=22, mean age: 10.18 ± 1.10 years), the ADHD with sports game (SG) (AWSG, n=16, mean age: 9.38 ± 0.96 years), and the typically developing (TD) control group (CG, n=17, mean age: 8.94 ± 0.56 years). The AWRSE underwent a RSE intervention, while the other two groups participated in SG. The exercise intervention lasted for 8 weeks, with sessions held twice a week for 60 minutes each, at a moderate-to-vigorous-intensity (64–95% HRmax). All children in each group underwent pre-test and post-test, including height, weight, BMI, n-back, and 20mSRT. One-way analysis of variance (Ony-way ANOVA) and paired sample t-test were used to analyze inter- and intra-group differences respectively.

**Results:**

Before the intervention, children with ADHD exhibited a significantly lower VO_2_max compared to the TD children (p<0.05), and there was no significant difference in the other indicators between the groups (p>0.05). After the intervention, no significant inter-group differences were found across all indices for the three groups of children (p > 0.05). The AWRSE had significant improvements in the accuracy of 1-back task, Pacer (laps), and VO_2_max (p<0.05), with the level of CRF approaching that of TD children. A significant decrease in response time for the 1-back task was observed in the CG.

**Conclusion:**

An 8-week RSE intervention is an effective therapeutic approach for children with ADHD, significantly enhancing their WM and CRF.

## 1 Introduction

Attention Deficit and Hyperactivity Disorder (ADHD) is a neurodevelopmental disorder characterized by core symptoms of inattention and hyperactivity/impulsivity (1). Epidemiological surveys indicate that the global prevalence of ADHD is approximately 5.29% (2), with up to 7.2% of affected individuals being children and adolescents (3). The overall prevalence of ADHD among children in China is 5.6% (4). Among these children, 50% to 70% continue to exhibit symptoms of ADHD into adulthood (1). The core symptoms of ADHD are closely associated with deficits in Executive Function (EF) (5), with Working Memory (WM) being one of the primary contributors to these deficits (6). Approximately 80% to 85% of children with ADHD have WM impairments (7). The core symptoms of ADHD are related to a lag in the memory-updating mechanism of WM (6, 8). Additionally, poor physical fitness is one of the risk factors for an increase in ADHD symptoms during childhood, with Cardiorespiratory Fitness (CRF) being implicated in the manifestation of ADHD symptoms (9). The majority of studies have confirmed that children with ADHD have significantly lower levels of CRF compared to their healthy, same-aged peers, and this deficiency is generally consistent (10–12), unaffected by demographic factors such as gender, age, and BMI (13).

With the escalating prevalence of sedentary behavior among children, the association between CRF and cognitive function assumes increasing significance. Impaired CRF is linked to cognitive deficits (14). Several studies have demonstrated a positive correlation between CRF and WM in children with ADHD. Enhanced CRF in ADHD children is associated with reduced reaction time and increased P3 amplitude, particularly under high memory load conditions (15, 16). Furthermore, utilizing thresholds of CRF can aid in early identification and intervention for low WM performance in children (17), while also mediating the relationship between physical activity and academic achievement (18). Therefore, enhancing both CRF and WM holds immense importance for children with ADHD.

Currently, although the benefits of medication for ADHD in children may outweigh the risks (19), there remains a subset of ADHD patients who experience poor efficacy and even side effects following drug treatment (20). Furthermore, nonpharmacologic treatments have demonstrated limited long-term effectiveness beyond 24 months (21). Additionally, mainstream traditional treatment methods such as pharmacotherapy and behavioral therapy primarily target cognitive improvement and reduction of abnormal distracted behaviors in ADHD children (20), with no significant positive impact on their CRF (22). Considering ADHD as a chronic disease necessitates comprehensive management and treatment approaches (1); exercise intervention presents an alternative or adjunctive option to drug therapy without associated side effects (23). Therefore, long-term regular exercise intervention holds substantial research and practical value.

When considering the positive effects of long-term regular exercise intervention on children with ADHD, qualitative exercise characteristics may play a crucial role (24). Recent meta-analysis findings indicate that engaging in moderate-to-vigorous-intensity closed aerobic exercises over an extended period can significantly enhance WM in children with ADHD (25, 26). Rope skipping exercise (RSE) is a type of closed exercise that necessitates individuals to follow a predetermined movement pattern, typically within a consistent and stationary self-paced environment (26). Moreover, RSE requires high levels of physical fitness and cardiopulmonary function, while maintaining the individual’s heart rate within the middle-to-high-intensity range during the activity (27). Additionally, RSE is not only cost-effective and enjoyable but also more suitable for widespread implementation in limited spaces compared to common activities like walking or running (28). Existing studies have demonstrated that an 8-week RSE can improve CRF among special needs children and increase autonomic activation in the prefrontal cortex associated with WM (29, 30). Therefore, it is hypothesized that an 8-week RSE could positively impact both WM and CRF in children with ADHD. The objective of this study was to investigate whether an 8-week RSE would yield beneficial outcomes for WM and CRF among children with ADHD.

## 2 Methods

### 2.1 Participants

This research received approval from the Scientific Research Ethics Committee at Shanghai Sport University under reference number NO:102772022RT123. Written informed consent was obtained from all participating parents/guardians according to the requirements outlined in the Declaration of Helsinki. (**Supplementary Material Parental Informed Consent Form**) 

Participants in this study were recruited based on the following inclusion criteria (1): participants aged between 6 and 12 years (2); a diagnosis of ADHD according to the Diagnostic and Statistical Manual of Mental Disorders, Fifth Edition (DSM-5) (3); Intelligence Quotient (IQ) above 80, as assessed by the Chinese Wechsler Intelligence Scale for Children (C-WISC) (4); the capacity to comprehend, adhere to the research team’s instructions, and actively participate in the required examinations, assessments, and intervention procedures. Exclusion criteria were as follows (1): presence of other comorbid conditions, including intellectual disabilities (ID), autism spectrum disorder (ASD), or conduct/disruptive behavior disorders (2); history of cerebral trauma or brain damage, or current medical conditions such as cardiovascular disease, asthma, diabetes, or hypertension (3); use of medications unrelated to ADHD treatment, including sedatives and other psychotropic drugs (4); absence from more than one-third of the scheduled exercise intervention sessions.

Fifty-five children diagnosed with ADHD and 27 typically developing (TD) children (age range: 6–12 years) were recruited from two primary schools in Shanghai for this study. All children with ADHD recruited for the study were diagnosed by professional psychiatrists according to the DSM-5. The specific diagnostic process included reviewing past medical history, clinical observation, and interviews, with the Conners Child Behavior Scale serving as Supplementary Material. Among them, seven children with ADHD were found to have comorbid conditions such as ID or ASD upon diagnosis by the professional psychiatrist, or were found to have diseases such as heart disease or asthma after reviewing their past medical history, and were therefore excluded from the experiment. Additionally, ten children with ADHD and ten TD children were removed from the study due to withdrawal or absenteeism exceeding one-third of the total number of intervention sessions. Ultimately, 55 children (mean age: 9.56 ± 1.05 years, mean BMI: 18.49 ± 3.53) were included in the final experimental study, comprising 38 children with ADHD (27 boys and 11 girls) and 17 TD children (11 boys and 8 girls).

As shown in **Table 1**, the ADHD with RSE (AWRSE) consisted of 22 children with ADHD (mean age: 10.18 ± 1.10 years), the ADHD with sports game (AWSG) included 16 children with ADHD (mean age: 9.38 ± 0.96 years), and the control group (CG) comprised 17 TD children (mean age: 8.94 ± 0.56 years). The baseline Body Mass Index (BMI) levels were consistent across the three groups of children (F=0.759, p=0.473>0.05). However, children with ADHD were older than TD children, with the AWRSE’s age being significantly greater than that of the CG (F=10.452, p=0.000 < 0.05).

**Table 1:** Demographic characteristics of participants (M ± SD).

Groups	Age (years)	F	p-values	two-by-two comparison of p-values			BMI (kg/m²)	F	p-values
				①vs②	①vs③	②vs③			
AWRSE (n=22)	10.18 ± 1.10	10.452	0.000^**^	0.054	0.000^**^	0.274	19.18 ± 3.77	0.759	0.473
AWSG(n=16)	9.38 ± 0.96						18.28 ± 4.07		
CG (n=17)	8.94 ± 0.56						17.80 ± 2.56		
Overall (n=55)	9.56 ± 1.05						18.49 ± 3.53		

AWRSE, ADHD with Rope Skipping Exercise; AWSG, ADHD with Sports Games; CG, Control Group, ^*^ p<0.05, representative p values have significant differences; ^**^ p<0.01, represents p values with extremely significant differences, the same as below.

### 2.2 Procedures

**Figure 1:**
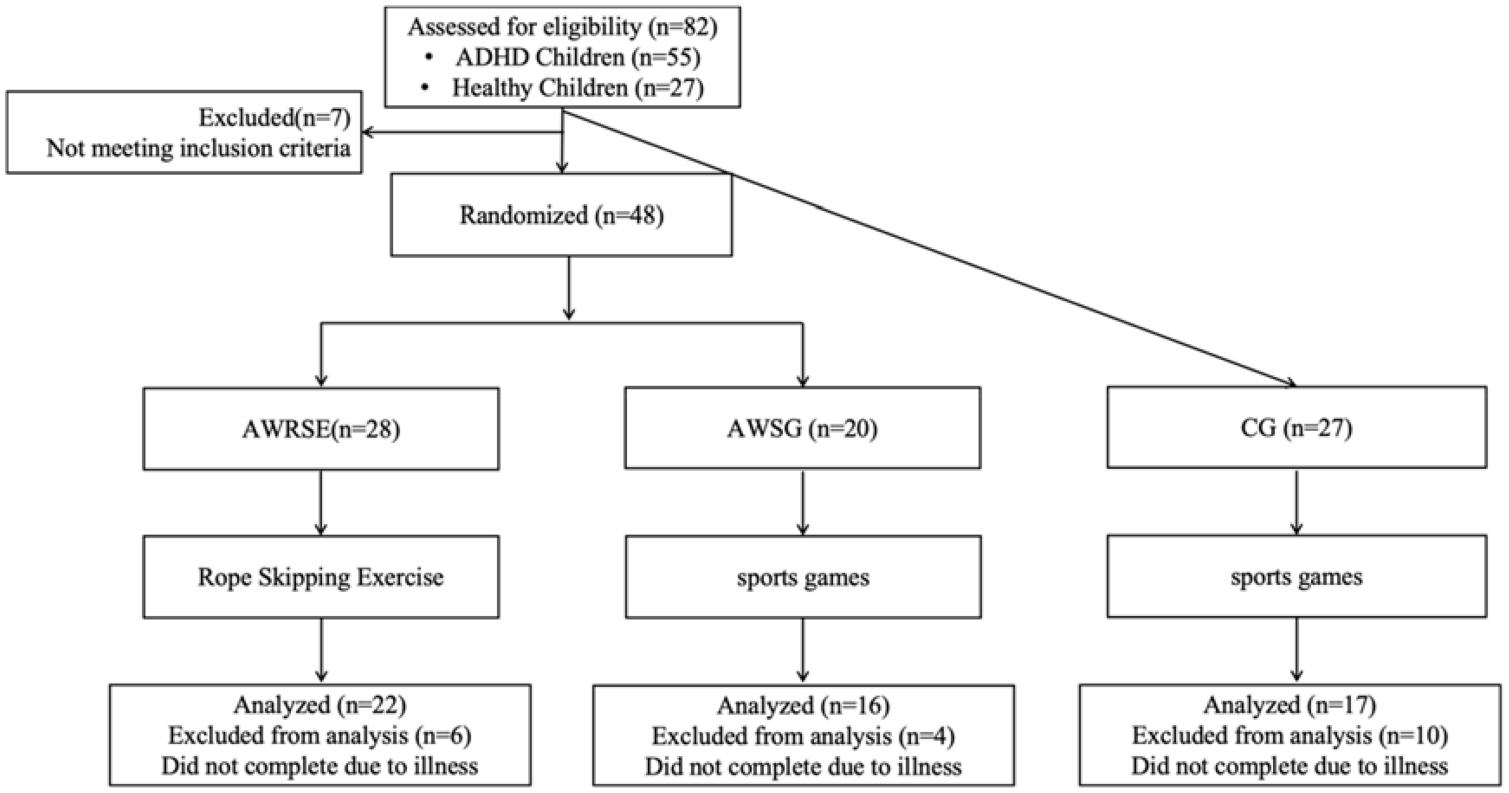
Study flow chart. AWRSE, ADHD with Rope Skipping Exercise; AWSG, ADHD with Sports Games; CG, Control Group, the same as below.

The exercise intervention process is illustrated in **Figure 1**. A 3×2 experimental design was employed, with the group factor (inter-subject factor) consisting of AWRSE, AWSG, and CG, and the time factor (intra-subject factor) comprising pre-test and post-test measurements. The entire experiment encompassed three stages: pre-test, intervention, and post-test. Initially, all three groups of children underwent a pre-test one week before the exercise intervention. Subsequently, an eight-week intervention period followed during which the AWRSE engaged in RSE while both AWSG and CG participated in sports games (SG). Finally, a post-test was conducted one week after the completion of the exercise intervention. Physical fitness assessments including height, weight, and BMI were performed in the school health care room; WM evaluations took place in the school computer room; CRF measurements were carried out on the school playground. Each participant’s testing procedure lasted approximately 30 minutes with breaks between each test.

The AWRSE participated in an 8-week intervention of RSE, conducted twice a week (on Tuesdays and Thursdays, from 16:30 to 17:30), with each session lasting 60 minutes. The intervention program was collaboratively developed by a rope skipping coach and a special education teacher. The program was implemented by an intermediate-level rope skipping coach with 3 years of teaching experience, assisted by three graduate students majoring in Adaptive Physical Education or Psychology. Each intervention session consisted of three parts: warm-up (10 minutes), rope skipping practice (40 minutes), and cool-down (10 minutes) (**Table 2**). The warm-up involved light jogging and joint exercises to increase body temperature, reduce muscle viscosity, and enhance joint mobility. The rope skipping practice included both unroped and roped exercises, all performed in a timed and relay format. The timed exercises consisted of 5 sets of 3 minutes each, with a 1-minute rest between sets. The relay exercises were conducted in teams of 4 to 5 children, with the team completing the most repetitions within the set time declared the winner. The cool-down involved static stretching.

**Table 2:** Weekly design of the 8-week RSE intervention for the ADHD.

Section		Exercise Content	Duration (Min)
Warm up		Jogging and joint exercises	10
rope skipping practice	1-2 weeks	And take off the foot and beat the leg, jump on the side of the body and jump on the front of the body	40
	3-4 weeks	Single jump rope, squat up jump rope, two step one shake run rope	
	5-6 weeks	Pad step jump rope, one step shake run rope, and foot low jump	
	7-8 weeks	Tandem with jump rope, timed relays	
Cool down		Static stretching	10

RSE, rope skipping exercise.

The remaining two groups (AWSG and CG) engaged in SG during the 8-week exercise intervention period, with two sessions per week, each lasting 60 minutes. The intervention was conducted by a physical education teacher with extensive teaching experience, assisted by three graduate students majoring in Adaptive Physical Education or Psychology. The content of the SG was designed by the physical education teacher, with each session comprising three different games to ensure exercise intensity, primarily conducted in the form of group relay races, including: relay running, relay crawling, and relay carrying. The difficulty of the games progressed from straight lines to curves, from obstacle-free to obstacle-inclusive, and from individual to team-based starts. Each SG lasted for 10 minutes, with a 3 to 5-minute rest period scheduled between games.

### 2.3 Measures

#### 2.3.1 Working memory (WM)

This study utilized the n-back task to assess the WM levels of the participating children, employing E-prime 3.0 software for the programming and administration of the letter n-back task. The evaluation metrics were response time (RT) and accuracy (ACC). Given the attention deficits associated with children with ADHD, reducing the difficulty of the n-back task is more appropriate for children with this disorder (31), and it has been confirmed that the n-back task remains an effective method for testing the WM of children with ADHD when simplified (32). In this study, we referred to existing research and used the two English letters (A and B), employing two memory load conditions (n=1, 2). The entire test comprised two subtasks: the 1-back and 2-back tasks (33). Each sub-task included eight practice trials followed by sixteen formal trials. Within each trial, letter stimuli were presented in a completely randomized order. ADHD children were instructed to press the mouse button promptly upon making correct judgments; otherwise, they were required to press the right mouse button instead. A blank screen lasting between 500–1000ms separated consecutive letter stimuli.

#### 2.3.2 Cardiopulmonary fitness (CRF)

In this study, the 20-meter Shuttle Run Test (20mSRT) was employed to assess the participants’ CRF, with the evaluation indicators being the total number of laps completed in Pacer (laps) and VO_2_max. Each lap covered a distance of 20 meters, and for every completed lap, one point was awarded. The Pacer (laps) score was then converted into VO_2_max using the Leger formula (34): VO_2_max = 31.025 + 3.238 × maximum speed - 3.248 × age + 0.1536 × age × maximum speed (where maximum speed = 8 + 0.5 × final intensity level). During testing, upon hearing the “start” signal, children were instructed to run from the starting point to the endpoint while gradually increasing their running speed in synchronization with the music rhythm. If a child discontinued due to physical exhaustion during testing, their result would be based on their last completed lap across the finish line. In cases where a child failed to complete two consecutive round trips within the designated time frame, testing would be terminated and their current number of laps minus two unfinished laps would be recorded as their final total number of laps completed.

### 2.4 Quality control of the experiment

This study ensured that the testers conducting both pre- and post-tests were consistent. Additionally, the study adhered to a double-blind experimental design, where the rope skipping coach and the physical education teachers were not informed of the presence of children with ADHD nor were they aware of the group assignments. Moreover, the participating children themselves were also unaware of their group allocations. To monitor exercise intensity during the intervention, heart rate was measured using Polar HR (Polar OH1 Optical Heart Rate Sensor), which was worn on the left upper arm (35). The maximum heart rate for each group of children was predetermined using Polar^®^ BHT TEAM SYSTEM with the formula HRmax=208–0.7× age (36). The target exercise intensity for this study was set at thirty minutes of moderate-to-vigorous-intensity exercise (64–95% HRmax).

### 2.5 Statistical analysis

The SPSS 26.0 software was utilized for data analysis. Before conducting statistical tests, the baseline data were assessed for normality and homogeneity of variance. For normally distributed or approximately normally distributed data, variance analysis was employed to examine group differences. In cases where the homogeneity of variance assumption was met, a one-way analysis of variance (ANOVA) followed by an LSD test was conducted for pairwise comparisons between groups. Alternatively, if the assumption of homogeneity of variance was violated, Welch’s ANOVA and Games-Howell test were used for *post hoc* comparisons between any two groups. Paired sample t-tests were performed to analyze within-group changes following exercise intervention, with Cohen’s d used as an effect size measure for t-tests (where 0.2≤d<0.5 represented a small effect size, 0.5≤d ≤ 0.8 denoted a medium effect size, and d>0.8 indicated a large effect size). Mean ± standard deviation (M ± SD) values were reported as descriptive statistics and p<0.05 served as the threshold for statistical significance.

## 3 Results

In this study, heart rate and duration of moderate-to-vigorous-intensity exercise were measured during the exercise intervention process. The results showed that during the experiment, the average heart rate of the AWRSE was 143.23 ± 4.44 beats per minute (71.30% of HRmax), with an average duration of moderate-to-vigorous-intensity exercise being 40.09 ± 4.63 minutes. Additionally, the average heart rate of the AWSG was 138.89 ± 6.53 beats per minute (68.99% of HRmax), with an average duration of moderate-to-vigorous-intensity exercise being 37.42 ± 4.93 minutes. The CG’s average heart rate was 143.60 ± 11.32 beats per minute (71.18% of HRmax), with an average duration of moderate-to-vigorous-intensity exercise being 38.28 ± 4.43 minutes. The above results indicate that the exercise intensity for all groups of children was controlled within the moderate-to-vigorous-intensity range.

### 3.1 Differences in working memory before vs. after intervention

As shown in **Table 3**, the ACC and RT for the 1-back and 2-back tasks of the three groups of children before the intervention met the assumption of homogeneity of variance (p > 0.05). The results of One-way ANOVA indicated no statistically significant differences between groups (p > 0.05). These results suggest that the WM of the three groups of children was at the same baseline level before the intervention. As shown in **Table 4**, the ACC and RT for the 1-back task, as well as the RT for the 2-back task, of the three groups of children after the intervention also met the assumption of homogeneity of variance (p > 0.05). The results of one-way ANOVA indicated no statistically significant differences between groups (p > 0.05). However, the ACC for the 2-back task did not meet the assumption of homogeneity of variance (p = 0.044 < 0.05), and Welch’s ANOVA was used instead, which also showed no statistically significant differences between the three groups (F = 0.670, p = 0.519). These results indicate that the WM of the three groups of children remained at the same level after the intervention.

**Table 3:** Difference in various indexes between each groups in pre-test.

Latitude	Indicators	AWRSE (n=22)	AWSG(n=16)	CG (n=17)	F	p-values	two-by-two comparison of p-values		
							①vs②	①vs③	②vs③
WM	ACC (%)								
	1-back	76.70 ± 20.06	79.30 ± 17.64	82.35 ± 13.66	0.494	0.613			
	2-back	71.02 ± 19.15	63.67 ± 21.07	70.59 ± 23.67	0.651	0.526			
	RT (ms)								
	1-back	1388.28± 643.50	1393.39 ± 633.35	1303.40 ± 496.53	0.124	0.883			
	2-back	1616.31 ± 765.09	1782.70 ± 1411.65	1962.43 ± 827.66	0.563	0.573			
CRF	Pacer (laps)	14.64 ± 5.61	14.56 ± 4.10	20.82 ± 9.40	3.279	0.051			
	VO_2_max (ml/kg/min)	42.28 ± 2.27	43.70 ± 2.39	45.80 ± 2.71	9.933	0.000^**^	0.083	0.000^**^	0.017^*^

BMI, Body Mass Index; WM, Working Memory; CRF, Cardiorespiratory Fitness; ACC, accuracy; RT, response time, the same as below.

**Table 4:** Difference in various indexes between each groups in post-test.

Latitude	Indicators	AWRSE (n=22)	AWSG(n=16)	CG (n=17)	F	p-values
WM	ACC (%)					
	1-back	86.36 ± 15.51	78.91 ± 17.66	87.87 ± 13.16	1.599	0.212
	2-back	76.99 ± 14.61	69.53 ± 23.37	76.84 ± 15.90	0.670	0.519
	RT (ms)					
	1-back	1152.61 ± 258.78	1233.92 ± 355.36	1125.14 ± 381.76	0.489	0.616
	2-back	1901.82 ± 857.05	1905.45 ± 950.15	1886.91 ± 695.83	0.002	0.998
CRF	Pacer (laps)	18.59 ± 9.98	15.38 ± 4.49	21.12 ± 17.63	1.519	0.236
	VO_2_max (ml/kg/min)	43.48 ± 3.35	43.84 ± 2.41	45.93 ± 4.73	1.689	0.201

**Figure 2:**
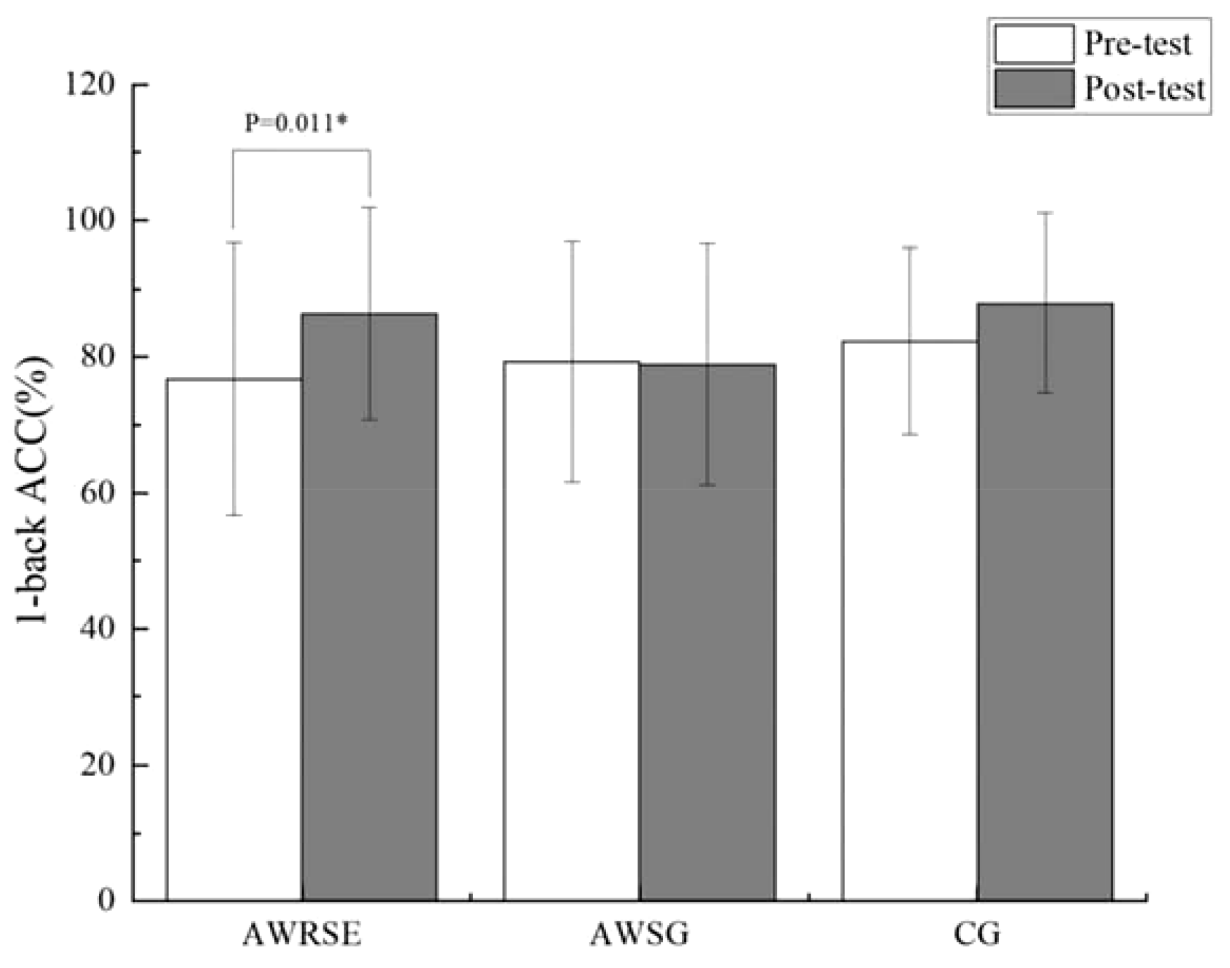
ACC in 1-back test to assess WM by group. ACC=accuracy, ^*^ p<0.05, representative p values have significant differences.

**Figure 3:**
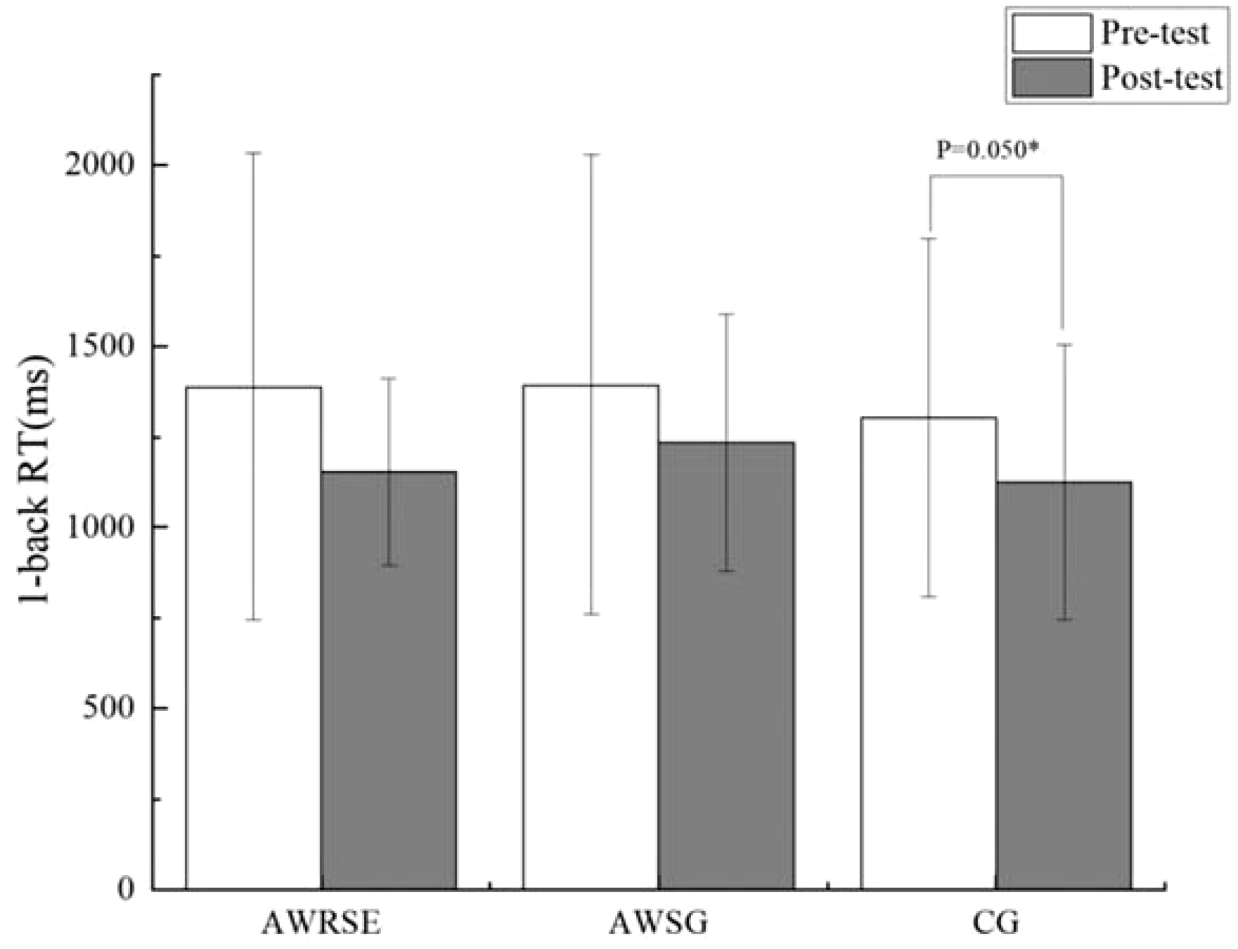
RT in 1-back test to assess WM by group. RT=response time, ^*^ p ≤ 0.05, representative p values have significant differences.

As shown in the results of the paired-samples t-test in **Table 5**, there was a significant increase in the ACC of the 1-back task for the AWRSE after the intervention compared to before, which was statistically significant (t = -2.793, p = 0.011 < 0.05, Cohen’s d = 0.595) (**Figure 2**). The CG showed a significant reduction in RT for the 1-back task, which was also statistically significant (t = 2.125, p = 0.050 ≤ 0.05, Cohen’s d = 0.515) (**Figure 3**). Although there were positive changes of varying degrees in the other indicators for each group, the differences within each group before and after the intervention were not statistically significant (p > 0.05). These results suggest that an 8-week RSE can significantly enhance the WM of children with ADHD, as reflected in the increased ACC of the 1-back task. In contrast, the two groups that received 8 weeks of SG showed only a significant reduction in RT for the CG’s 1-back task, leading to the suspicion that the positive effect may not be due to the SG itself but could be the result of other factors.

**Table 5:** Difference in WM between each groups in pre-test to post-test.

Indicators	AWRSE (n=22)		p-values	AWSG(n=16)		p-values	CG (n=17)		p-values
	Pre-test	Post-test		Pre-test	Post-test		Pre-test	Post-test	
ACC (%)									
1-back	76.70 ± 20.06	86.36 ± 15.51^*^	0.011^*^	79.30 ± 17.64	78.91 ± 17.66	0.943	82.35 ± 13.66	87.87 ± 13.16	0.168
2-back	71.02 **±** 19.15	76.99 ± 14.61	0.184	63.67 ± 21.07	69.53 ± 23.37	0.243	70.59 ± 23.67	76.84 ± 15.90	0.261
RT (ms)									
1-back	1388.28 **±** 643.50	1152.61 ± 258.78	0.085	1393.39 ± 633.35	1233.92 ± 355.36	0.325	1303.40 ± 496.53	1125.14 ± 381.76^*^	0.050^*^
2-back	1616.31 **±** 765.09	1901.82 ± 857.05	0.105	1782.70 ± 1411.65	1905.45 ± 950.15	0.697	1962.43 ± 827.66	1886.91 ± 695.83	0.713

### 3.2 Differences in cardiorespiratory fitness before vs. after intervention

**Figure 4:**
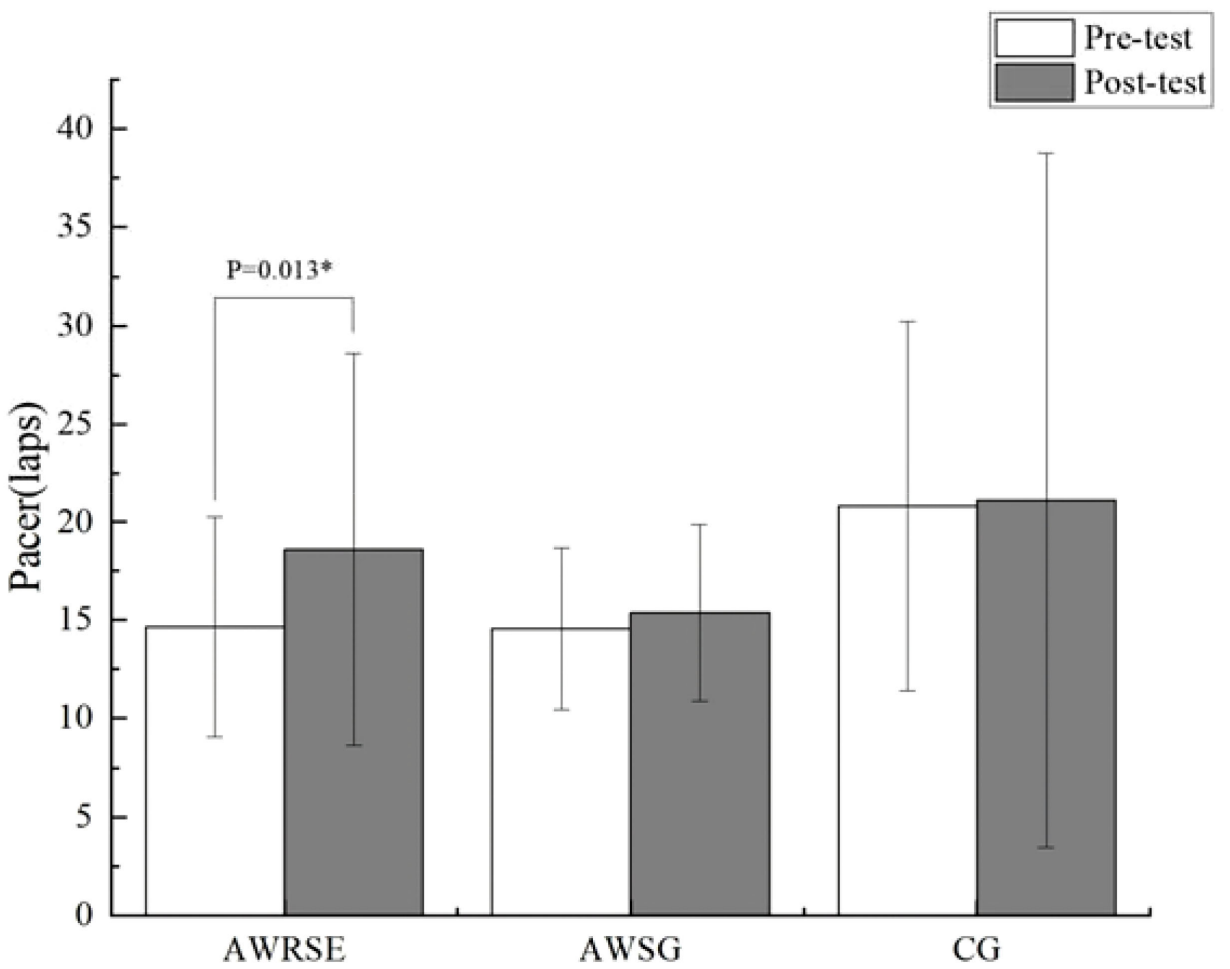
Laps in Pacer to assess CRF by group. ^*^ p<0.05, representative p values have significant differences.

**Figure 5:**
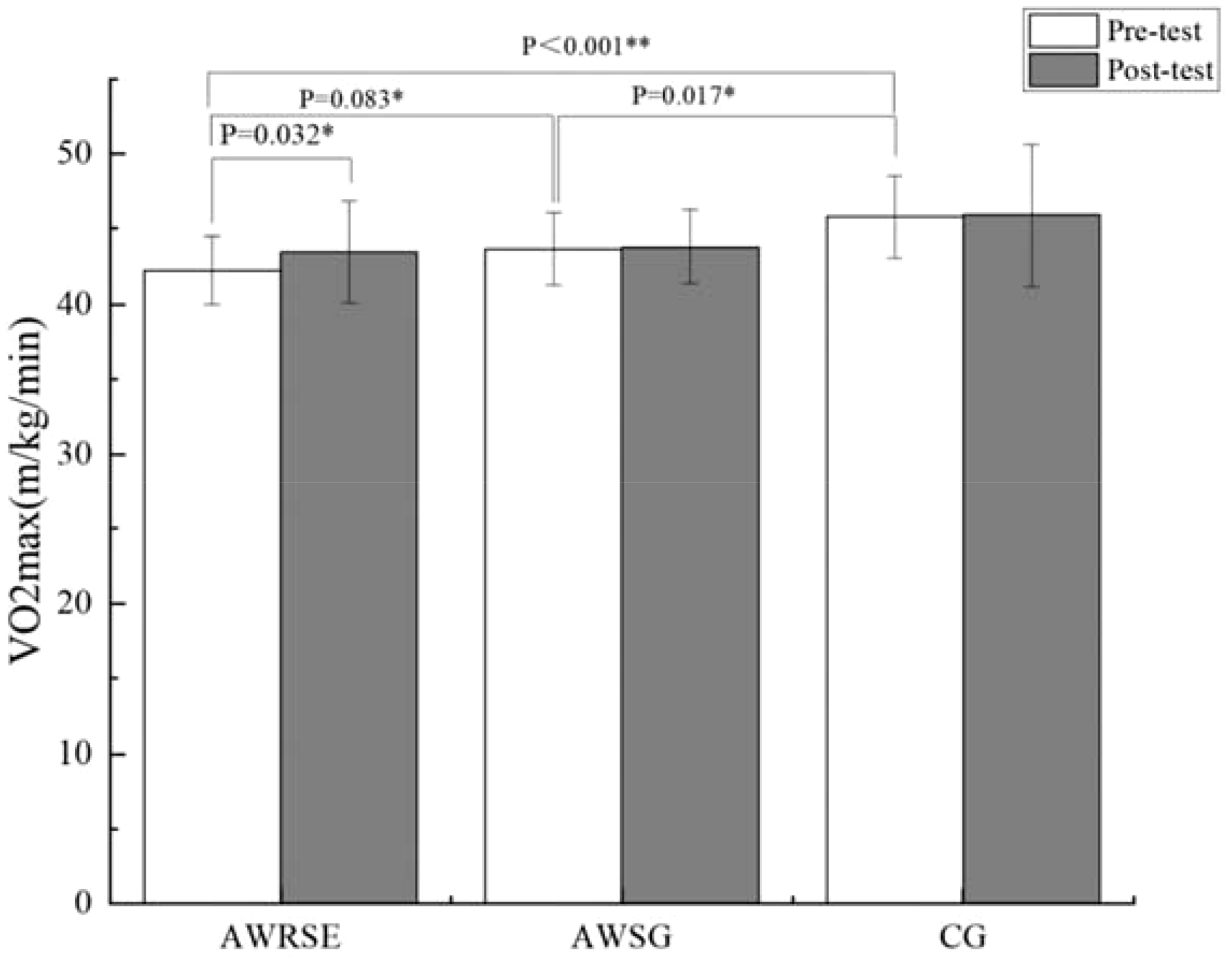
VO_2_max to assess CRF by group. ^*^ p<0.05, representative p values have significant differences, ^**^ p<0.01, represents p values with extremely significant differences.

As shown in **Table 3**, the Pacer (laps) of the three groups of children before the intervention did not meet the assumption of homogeneity of variance (P = 0.009 < 0.05), and Welch’s ANOVA was used, which showed no statistically significant differences between the groups (F = 3.279, p = 0.051 > 0.05) (**Figure 4**). The VO_2_max of the three groups of children before the intervention met the assumption of homogeneity of variance (p = 0.632 > 0.05), and one-way ANOVA revealed statistically significant differences between the groups (F = 9.933, p = 0.000). Further LSD testing indicated that there was a statistically significant difference between the AWRSE and the CG (p = 0.000 < 0.05), as well as between the AWSG and the CG (p = 0.017 < 0.05). The VO_2_max of both groups of children with ADHD was lower than that of the TD children, and there was no statistically significant difference between the two groups of children with ADHD (p = 0.083 > 0.05) (**Figure 5**). In summary, before the intervention, the CRF of the two groups of children with ADHD was comparable but significantly lower than that of the TD children. As shown in **Table 4**, the Pacer (laps) (p = 0.001 < 0.05) and VO_2_max (p = 0.032 < 0.05) of the three groups of children after the intervention did not meet the assumption of homogeneity of variance, and Welch’s ANOVA showed no statistically significant differences between the groups for either Pacer (F = 1.519, p = 0.236) or VO_2_max (F = 1.689, p = 0.201). These results suggest that after 8 weeks of RSE and SG, the CRF of children with ADHD improved and approached the level of TD children.

As shown in the paired-samples t-test results in **Table 6**, there was a significant increase in the Pacer (laps) of the AWRSE after the intervention compared to before, which was statistically significant (t = -2.731, p = 0.013 < 0.05, Cohen’s d = 0.582) (**Figure 4**). At the same time, the VO_2_max of the AWRSE also significantly increased, with a statistically significant difference (t = -2.304, p = 0.032 < 0.05, Cohen’s d = 0.491) (**Figure 5**). Although the Pacer (laps) and VO_2_max of the AWSG and the CG both increased, the differences were not statistically significant (p > 0.05). These results suggest that 8 weeks of RSE had a greater impact on improving the cardiorespiratory fitness of children with ADHD compared to SG.

**Table 6:** Difference in CRF between each groups in pre-test to post-test.

Indicators	AWRSE (n=22)		p-values	AWSG(n=16)		p-values	CG (n=17)		p-values
	Pre-test	Post-test		Pre-test	Post-test		Pre-test	Post-test	
Pacer (laps)	14.64 ± 5.61	18.59 ± 9.98^*^	0.013^*^	14.56 ± 4.10	15.38 ± 4.49	0.085	20.82 ± 9.40	21.12 ± 17.63	0.933
VO_2_max (ml/kg/min)	42.28 ± 2.27	43.48 ± 3.35^*^	0.032^*^	43.70 ± 2.39	43.84 ± 2.41	0.333	45.80 ± 2.71	45.93 ± 4.73	0.885

## 4 Discussion

WM is a crucial component of EF, and children with ADHD often exhibit deficits in the core mechanism of WM—memory updating, and are typically unable to effectively control and allocate cognitive resources (8). In the present study, we employed the n-back task for assessment and unexpectedly found that the baseline levels of WM were consistent across all groups of children, indicating that the WM levels of children with ADHD were comparable to those of TD children. The discrepancy with existing research findings may be attributed to age differences among the participants. In this study, children with ADHD were older than TD children (**Table 1**). Current research suggests that the WM of children with ADHD improves with age (37), leading to the hypothesis that older children with ADHD may have a developmental level of WM equivalent to that of younger TD children. Additionally, inconsistencies in inclusion/exclusion criteria may also account for the differences in results. Firstly, the children with ADHD included in this study were free from comorbid conditions, such as ID or ASD, whereas existing studies have included children with ADHD who have such comorbidities (33, 38). Secondly, medication could also be a contributing factor to the differences in results. The EF of children with ADHD can be improved with stimulant medication, and there is no significant difference compared to TD children. This study allowed children to take their prescribed ADHD medication, whereas existing studies either prohibited medication intake 24 hours before testing or included only children who had never received any medication for ADHD (33, 38). In summary, the discrepancies in results between this study and existing research require further investigation for validation. However, the fact that the WM of the three groups of children was at the same baseline level enhances the comparability and credibility of the findings in this study.

One objective of this study was to investigate whether an 8-week RSE could enhance the WM of children with ADHD. The findings revealed that after the 8-week RSE, children with ADHD demonstrated a significant improvement in the ACC of the 1-back task. This is in line with existing research, such as the study by Hattabi et al. (39), which conducted a 12-week moderate-intensity aerobic exercise intervention on 40 elementary school children with ADHD and found that aquatic exercise significantly improved their WM. Current research indicates that closed motor skills, predominantly aerobic activities like RSE, are the most effective for enhancing the WM of individuals with ADHD (26). From the perspective of motor skill practice, activities that demand high coordination and require substantial attention are more effective in improving children’s WM (40, 41). The 8-week RSE in this study included various forms of unroped and roped jumping with timed practice, which are high coordination demand motor skill activities. Consequently, this necessitates that children with ADHD constantly engage their cognitive resources during the RSE to ensure the coordination of upper and lower limbs with the rope. Analyzing the mechanism of impact, RSE can improve WM by promoting neurotransmitters, such as catecholamines (23), which are similar to the mechanism of action of stimulant medications used to treat ADHD (42). It also enhances the secretion of dopamine, brain-derived neurotrophic factor (BDNF), and increases cerebral blood flow (43). Moreover, multiple neuroimaging studies have shown that aerobic exercises like RSE can alter brain plasticity, thereby improving the WM of children with ADHD. Functional magnetic resonance imaging (fMRI) studies have confirmed that after an 8-week RSE, there was a significant increase in the activation level of brain regions associated with WM in children with ADHD, such as the left middle frontal gyrus, right superior frontal gyrus, and right posterior cingulate cortex (30). Functional near-infrared spectroscopy (fNIRS) research has found that aerobic exercise can regulate known hypoactive areas in the brains of individuals with ADHD, such as the junction of the temporal and parietal lobes and the middle and lower parts of the frontal lobe (44). Additionally, resting electroencephalogram (EEG) studies have confirmed that after 8 weeks of aerobic exercise, children with ADHD have a smaller θ/α ratio in brain areas related to WM, such as the frontal and central brain regions (45). However, children with ADHD and TD children who underwent the same 8-week SG showed different intragroup changes in WM, with only the TD children showing a significant decrease in RT for the 1-back task. This suggests that the differences in results are not due to the 8-week SG but other potential factors. Existing research indicates that children with ADHD have motivational deficits and negative attitudes toward physical activity (46, 47), leading to lower participation rates in sports activities compared to TD children (48). This is consistent with the monitoring results from the Polar HR sensor, which showed that during the SG, children with ADHD had lower average heart rates and less time spent in moderate to high-intensity exercise compared to TD children. The reason for this may be related to the lower participation and compliance of children with ADHD in the SG compared to TD children. In summary, the 8-week RSE in this study was more effective than the SG and had a positive impact on the WM of children with ADHD.

Another objective of this study was to explore whether an 8-week RSE could improve the CRF of children with ADHD. CRF comprehensively reflects the body’s ability to intake, transport, and utilize oxygen, and it is an important dimension of physical fitness (49). The experimental results found that after the 8-week RSE, both the Pacer (laps) and VO_2_max of children with ADHD significantly improved. This is consistent with existing research findings, such as the study by Lee et al. (50), which conducted a 12-week moderate-to-vigorous-intensity exercise intervention on 12 elementary school children with ADHD and found that a comprehensive exercise intervention, including RSE, significantly enhanced the CRF of children with ADHD. Furthermore, there were no significant inter-group differences in Pacer (laps) and VO_2_max after the intervention among the three groups of children, the AWRSE showing a more significant improvement in Pacer, closer to the level of TD children. This experimental result is in line with the findings of Miguel et al. (51), which showed a positive correlation between participation in organized sports and changes in Pacer (laps). The 8-week RSE participated in by the AWRSE in this study had strict requirements for practice and rest time, which is a more organized form of exercise compared to SG. In this study, the RSE involved more timed practice, and the simplicity and repetitiveness of this motor skill practice process made it easier for children with ADHD to master and automate, thus allowing them to maintain the prescribed exercise intensity (23). Existing research indicates a significant dose-response relationship between CRF and the volume of moderate-to-vigorous physical activity (MVPA) (52). In this study, the average duration of moderate-to-vigorous-intensity exercise during the 8-week RSE for the AWRSE was 40.09 ± 4.63 minutes, which is approximately an increase of about 80 minutes of MVPA per week. Additionally, as an important indicator for evaluating CRF, the increase in VO_2_max with exercise is mainly due to the increase in the body’s maximum cardiac output (53). Therefore, in this study, the significant increase in VO_2_max after the 8-week RSE in the AWRSE may be since long-term regular aerobic exercise can significantly increase the left ventricular stroke volume (stroke volume), thereby leading to an increase in maximum cardiac output (54).

Ultimately, combining the indicators of WM and CRF for discussion, this study found that an 8-week RSE had a significant positive impact on both the WM and CRF of children with ADHD, as evidenced by significant improvements in the ACC of the 1-back task, Pacer (laps), and VO_2_max. These intragroup change results are consistent with those of Drollette et al. (16), where children with higher CRF demonstrated higher ACC in the 1-back task. However, there were some differences in the results; in this study, the ACC for the 2-back task in the AWRSE increased after the intervention compared to before, but the difference was not significant. This does not align with the expectation that when the memory load increases (2-back), children with higher CRF would also show more accurate target detection and discrimination abilities (16, 55). The possible reason for this discrepancy in results may be a ceiling effect in the WM test of this study, which did not accurately reflect the WM deficits of children with ADHD (56). It is precisely because of the age differences among the participants and the different inclusion/exclusion criteria that the children with ADHD in this study showed a preference in the n-back task, with their baseline WM levels being comparable to those of TD children. Furthermore, although the CRF of children with ADHD significantly improved after the 8-week RSE, and the statistical results showed no intergroup differences with TD children, the descriptive statistical results of their Pacer (laps) and VO_2_max data were still lower than those of TD children. Children with ADHD, as a special group attending regular classes, have significant importance in comparing their various abilities with those of TD children. This is crucial for their ability to integrate into the class community and participate in social activities. Subsequent considerations include extending the intervention period or increasing the intervention frequency for verification, attempting to find the most suitable combination of exercise dosage effects, to have a more positive impact on children with ADHD and to bring them closer to the levels of TD children.

## 5 Conclusion

After an 8-week RSE for children aged 6–12 years with ADHD in primary school, significant improvements were observed in both WM and CRF. Although the level of CRF approached that of TD children, a slight disparity remained. Therefore, RSE is deemed a suitable physical activity for children with ADHD.

## 6 Limitations

While this study has certain significance and contributions to understanding the impact of exercise interventions on the WM and CRF of children with ADHD, it is not without limitations. Firstly, the sample size included in this study was relatively small, and it was found that children with ADHD did not exhibit significant deficits in WM compared to TD children. Subsequent studies should verify these findings by adjusting the inclusion/exclusion criteria (particularly regarding comorbidities and medication use) and by expanding the sample size. Secondly, although the number of males in both the ADHD and TD control groups was greater than the number of females, the study did not analyze the results from the perspective of gender as a variable. Future research should consider the impact and interpretation of gender variables on the study outcomes. Additionally, the initial design of this study focused on the overall impact of RSE on the ADHD population. To achieve better exercise intervention outcomes, future research should consider examining the ADHD subtypes as a variable to further explore potential differences in the effects of exercise intervention on different ADHD subtypes.

## Data availability statement

The original contributions presented in the study are included in the article/supplementary material. Further inquiries can be directed to the corresponding author.

## Ethics statement

The studies involving humans were approved by the Ethics Committee of Shanghai University of Sport. The studies were conducted in accordance with the local legislation and institutional requirements. Written informed consent for participation in this study was provided by the participants’ legal guardians/next of kin.

## Author contributions

ZH: Data curation, Methodology, Software, Writing – original draft, Writing – review & editing. LL: Data curation, Investigation, Methodology, Software, Writing – review & editing. YL: Conceptualization, Project administration, Supervision, Writing – review & editing, Investigation. JM: Data curation, Formal analysis, Methodology, Writing – review & editing. XW: Methodology, Writing – review & editing, Conceptualization, Project administration, Supervision.
