# Supplementary material for: Effects of rope skipping exercise on working memory and cardiorespiratory fitness in children with attention deficit hyperactivity disorder
Source: Front Psychiatry. 2024 May 23;15:1381403. doi: 10.3389/fpsyt.2024.1381403 (PMC11153777; doi:10.3389/fpsyt.2024.1381403)
Supplement: Supplementary file 1 [file Presentation_1.pdf]

Parental Informed Consent Form

Dear Parents:

Hello! We would like to invite your child to join us at the "Happy Movement Camp". The camp will take place in several primary schools in Yangpu District, Shanghai, and we are committed to promoting the cognitive development of school-age children through sports development programmes. We have initially designed a 60-minute sports development programme that will run twice a week (Tuesdays and Thursdays from 4:30-5:30pm) for a period of 8 weeks. In the content design of the programme, we have designed a rich, interesting and targeted jump rope exercise content for the children.

In order to be able to understand the dynamic changes in the child's situation, timely adjustments, design a better quality, detailed sports development programme content, such as: the choice of exercise movements and the arrangement of the number of sets of exercises, and ultimately to make the children participating in the camp to obtain greater benefits, we need to be in the process of the camp on your child before and after the 2 tests, mainly including the following content: Body shape (height, weight and BMI) and Executive function (working memory) and Cardiorespiratory fitness.

We hereby promise that all the contents of the above tests are free of any side effects. In addition, the participants of the tests and programme have undergone relevant professional training and the following principles will be followed in the process:

Voluntary participation (participation in tests is completely voluntary and can be terminated at any time by changing your mind; refusal to participate will not negatively affect your child's future academic life).

Safety (Tests will be done to protect your child's health and safety as the first guideline and will not harm your child's health.)

Privacy protection (It is guaranteed that the personal information of children will not be disclosed, and it is promised that all the data collected, the informed letter will be placed completely separately, the photos and videos will be coded).

Return of Informed Consent

I have read the informed consent form in detail.

Agree with the child's participation☐

Disagree with the child's participation☐

Child's name:__________________

Parent Signature:________________

Contact number:________________

Date:________________

Shanghai University of Sport
